# High-throughput screening of the *Plasmodium falciparum* cGMP-dependent protein kinase identified a thiazole scaffold which kills erythrocytic and sexual stage parasites

**DOI:** 10.1038/s41598-019-42801-x

**Published:** 2019-05-07

**Authors:** Maria Penzo, Laura de las Heras-Dueña, Lydia Mata-Cantero, Beatriz Diaz-Hernandez, Maria-Jesus Vazquez-Muñiz, Sonja Ghidelli-Disse, Gerard Drewes, Elena Fernandez-Alvaro, David A. Baker

**Affiliations:** 1 GSK Global Health, Severo Ochoa 2, Tres Cantos 28760 Madrid, Spain; 2 Faculty of Infectious and Tropical Diseases, London School of Hygiene & Tropical Medicine, London, WC1E 7HT United Kingdom; 3 Cellzome GmbH, a GlaxoSmithKline Company, Meyerhofstrasse 1, 69117 Heidelberg, Germany

**Keywords:** High-throughput screening, Malaria

## Abstract

Antimalarial drug resistance compels the quest for new compounds that target alternative pathways to current drugs. The *Plasmodium* cyclic GMP-dependent protein kinase (PKG) has essential functions in all of the major life cycle developmental stages. An imidazopyridine PKG inhibitor scaffold was previously shown to clear *P. falciparum* infection in a rodent model *in vivo* and blocked transmission to mosquitoes providing proof of concept for this target. To find new classes of PKG inhibitors to serve as alternative chemical starting points, we performed a high-throughput screen of the GSK Full Diversity Collection using recombinant *P. falciparum* PKG. We developed a robust enzymatic assay in a 1536-well plate format. Promising compounds were then tested for activity against *P. falciparum* asexual blood stage growth, selectivity and cytotoxicity. By using a scoring system we selected the 66 most promising PKG inhibitors (comprising nine clusters and seven singletons). Among these, thiazoles were the most potent scaffold with mid-nanomolar activity on *P. falciparum* blood stage and gamete development. Using Kinobeads profiling we identified additional *P. falciparum* protein kinases targeted by the thiazoles that mediate a faster speed of the kill than PKG-selective compounds. This scaffold represents a promising starting point to develop a new antimalarial.

## Introduction

Malaria is a mosquito-borne protozoan infection caused by *Plasmodium* parasites. Five species are known to cause disease in humans (*P. falciparum*, *P. vivax*, *P. ovale*, *P. malariae* and *P. knowlesi*), amongst which *P. falciparum* is by far the most deadly. Infection starts when a female *Anopheles* mosquito injects *Plasmodium* sporozoites into the skin from where they reach the bloodstream during a blood meal. Sporozoites then travel to the liver within 15–30 minutes where they infect hepatocytes. Here they develop into liver schizonts through asexual multiplication to generate thousands of infective merozoites. These are then released into the bloodstream and invade red blood cells where they again undergo asexual replication. Following red blood cell invasion, merozoites develop into rings, trophozoites and multinucleated schizonts, each releasing up to 32 merozoites. This asexual blood phase causes all the clinical symptoms of malaria. A small proportion of merozoites develop into sexual precursor cells called gametocytes, which can be transmitted after a period of maturation lasting ~10 days to a mosquito following a blood meal. Once in the mosquito, the surrounding membranes of mature gametocytes rupture releasing male and female gametes, which fuse to form the zygote, the motile ookinete and finally the oocyst, where asexual replication takes place with thousands of sporozoites liberated that migrate to the salivary glands to be transmitted to a human host thereby completing the life cycle.

Malaria symptoms include high fever episodes, chills, lethargy and complications that can lead to coma and death. Fortunately, significant global health investments contributed to the observed decrease in malaria mortality of over 60% between 2000 and 2016^1^; but still an estimated 435,000 people died of malaria in 2017, 61% of those being children under the age of five^2^. The improvement in malaria morbidity and mortality rates is threatened by the observed increase in parasite resistance to all antimalarial drugs^3–5^ and mosquito resistance to insecticidal agents^6^. Together with complementary control/elimination measures, there is a clear need for new drugs with distinct modes of action for inclusion in combination treatments to counter resistance generation and strengthen control and elimination programs.

The drug discovery cascade begins with a screening process, which identifies hit compounds whose properties (such as activity, solubility and safety) are optimized in hit-to-lead and lead optimisation phases to deliver candidates that enter clinical development, a very small percentage of which become drugs. Target-based or phenotypic screens are the two approaches used to identify hits that will populate the pre-clinical and clinical pipeline. Target-based approaches rely on the identification of essential targets for parasite survival and development of a high-throughput assay to identify compounds that inhibit the activity of the target. Hits are then progressed to parasite growth inhibition assays and beyond. The advantages of the target-based approach include allowing more efficient compound optimisation and toxicology prediction is far more accurate. However, the target-based approach has historically been disappointing for the discovery of new antimalarials, mainly because of the lack of strongly validated targets and the challenges to identify compounds where target-based activity correlates with cell-based activity. On the other hand, malaria parasite phenotypic screens select compounds that inhibit parasite growth therefore identifying relevant targets in their biological context. The disadvantages are the more challenging and less rational structure-activity relationship (SAR), due to a lack of knowledge of the target, and the uncertainties regarding the therapeutic profile. Even with these caveats, phenotypic screening is the main approach that the antimalarial community has pursued in recent years^7–10^. Recent advances in *Plasmodium* genetic manipulation methodology and knowledge of parasite biology provide a new opportunity for the target-based approach.

One of the enzymes being explored by a target-based approach is the *Plasmodium* cyclic GMP-dependent protein kinase (PKG). This is a serine/threonine protein kinase essential in all of the key stages of the parasite’s life cycle^11–21^. PKG inhibitors in conjunction with an engineered inhibitor-insensitive mutant line have been used extensively in *P. falciparum* and *P. berghei* to determine the role of the kinase throughout the life cycle. For example, PKG in the rodent malaria species *P. berghei* (PbPKG) was shown to be involved in hepatocyte infection by sporozoites. PbPKG regulates sporozoite motility through the modulation of protein release from apical organelles called micronemes^20^. In addition, evidence from a conditional knockout approach suggested that PbPKG regulates a late step in liver stage development^15,20^. In the *P. falciparum* asexual blood stage, schizont rupture is dependent on PKG^16^. Merozoite egress is a rapid, tightly controlled process that involves multiple proteins. PfPKG was shown to regulate the discharge of the protease PfSUB1 from the exonemes and its subsequent proteolytic cleavage of MSP1 and the SERA family^17,22^. PKG inhibition also blocked release of AMA1 from micronemes^17^ and prevented merozoite invasion of red blood cells^19^. In the mosquito, gametogenesis^13^ and ookinete motility are also specifically blocked by PKG inhibitors^14,18^. A global phosphoproteomic approach identified 69 *P. falciparum* schizont proteins involved in cell signalling, proteolysis, ion transport, transcriptional regulation, protein export and chromatin regulation that are phosphorylated in a PKG-dependent manner^19^. PKG also has a key role in controlling the levels of intracellular calcium through regulation of phosphoinositide metabolism in *P. falciparum* and *P. berghei*^18^.

Several features of the malaria parasite PKG differ from mammalian PKGs, including the presence of two additional conserved cGMP-binding sequence motifs, where one of these does not bind cGMP but is essential for activity^23,24^. Another peculiarity is the presence of a threonine residue in the so-called gatekeeper position. This renders a hydrophobic pocket in the ATP-binding site accessible to a class of ATP-competitive inhibitors^25,26^. Most mammalian Ser/Thr kinases have a larger residue in the gatekeeper position, meaning that inhibitors that utilize the hydrophobic pocket cannot bind^27^. Importantly, both human PKGs have large gatekeeper residues and are insensitive to this class of inhibitor. The small gatekeeper residue has been exploited to generate PKG-selective inhibitors^11^. Substitution of the PfPKG gatekeeper threonine residue with glutamine (with a bulkier side chain), prevents access of the inhibitor to the hydrophobic gatekeeper pocket which adjoins the ATP-binding site^13^. This chemical genetic approach proved an excellent tool to define off-target effects on parasites, as well as to study PKG-dependent processes in the parasite^13,16,18,19^.

A recent medicinal chemistry study used an imidazopyridine series to provide *in vivo* proof of concept that PKG inhibition can clear malaria infection in a *P. falciparum* rodent model as well as block transmission to mosquitoes^11^. Some imidazopyridines have given positive Ames tests^28^, which raises issues from the drug discovery point of view. Therefore we decided to look for additional PKG inhibitor scaffolds using high-throughput screening (HTS), to provide additional starting points for an antimalarial therapy based on PKG inhibition.

## Results

### High-throughput screening identifies potent PKG inhibitors

**Figure 1:**
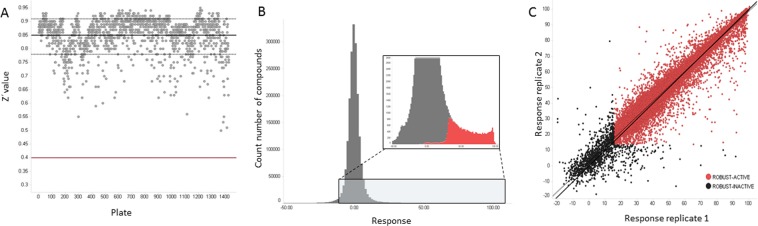
Performance metrics of HTS on PfPKG. (**A**) Plate number is plotted against the resulting Z′^61^. The red line corresponds to the minimum required value to deem the assay of good quality and the thick black line corresponds to the mean value of 0.85. (**B**) Distribution of the percentage inhibition in the 10 μM concentration assay for the 1.7 million compounds. Compounds were selected above the cut-off of 30% inhibition (in red). (**C**) Correlation of the confirmation step tested in duplicate against WT PfPKG.

To identify new PKG inhibitor scaffolds, the 1.7 million GlaxoSmithKline Full Diversity collection was tested at a single final concentration of 10 μM against recombinant full length wild type (WT) PfPKG using a Kinase Glo-based assay (Promega). The results gave a robust Z′ mean value of 0.85. At an average statistical cut-off of 30%, 20,174 primary hits were identified with an overall hit rate of 1%. Representative HTS performance metrics are shown in Fig. 1. Fresh samples of these primary hits were tested in duplicate against WT PfPKG and also against human PKGIα (HuPKGIα) (Supplementary Fig. 1) at a final concentration of 10 μM (confirmation step).

**Figure 2:**
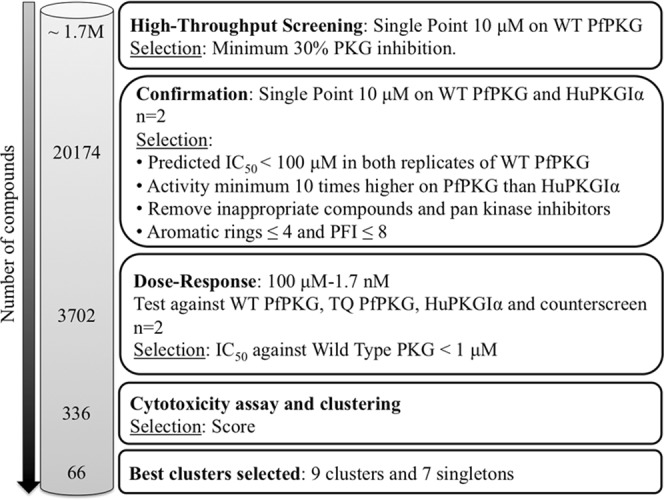
Progression cascade of the high-throughput screen. The number of technical replicate assays is indicated (n). Individual steps of the cascade are described in the text.

The confirmed hit list was narrowed down (Fig. 2) after the application of the following filters: predicted WT IC_50_ PfPKG lower than 100 µM (Predicted IC_50_ = [(100-%Inhib)/%Inhib]concentration (μM)); minimum 1 log difference in predicted IC_50_ for *Plasmodium* PKG over HuPKGIα, absence of undesirable structural features (such as electrophiles, peroxides, Michael acceptors) and reduced physicochemical risk (maximum 4 aromatic rings and PFI lower than 8)^29^. This procedure resulted in a subset of 6,086 hits. The application of a GSK internal program to choose a set of compounds that are diverse in chemical space resulted in a final list of 3,702 compounds that were progressed to dose response studies. Dose response assays were performed in duplicate on WT PfPKG, HuPKGIα and a mock assay performed in the absence of enzyme (the counterscreen). Comparison of the PfPKG and HuPKGIα IC_50_ values gave information on selectivity and the counterscreen identified luminescent false positive compounds (see Materials and Methods). Cytotoxicity assays were also performed using a human hepatoma cell line (HepG2)^30^. A total of 336 compounds gave IC_50_ values of <1 μM using WT PfPKG. This set of compounds was clustered by similarity using a complete-linkage algorithm and a threshold of 0.55^31^. A total of 79 clusters and 54 singletons were obtained.

**Figure 3:**
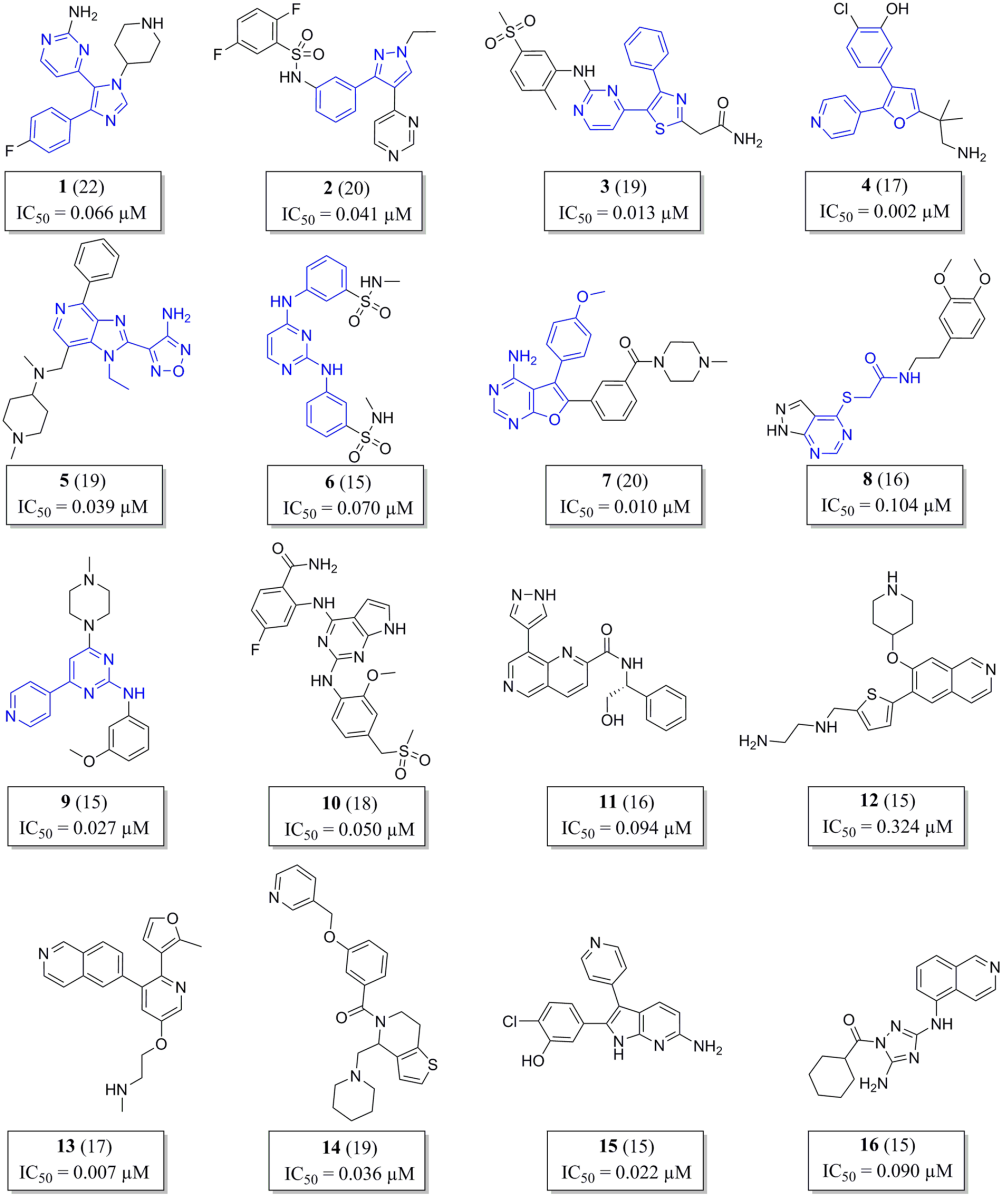
Prioritized compounds in their respective cluster class. Of the 66 prioritised compounds, the structure of the most scored compound in each cluster is shown, the common scaffold moiety across cluster representatives is coloured in blue. Each compound’s score is shown in parentheses and the IC_50_ value corresponds with WT PfPKG activity.

In order to prioritize the chemotypes, we developed a scoring system (Table 1) that considers potency, selectivity, cytotoxicity, ligand efficiency, lipophilic ligand efficiency, promiscuity and cluster size. The scoring system aimed to filter out as early as possible in the hit selection process compounds with selectivity/toxicity issues. Activity against WT PfPKG of <0.1 μM was given the highest weight (a score of 6) and a score of 3 or 1 was assigned based on the other parameters examined (Table 1). Compounds scoring more than 15 points were selected, resulting in 66 compounds with a balanced hit profile belonging to 9 clusters and 7 singletons. The score of each compound and the structures of the most active compound in each cluster and singleton structures are shown in Fig. 3.

**Table 1:** Scoring system used to prioritize chemotypes.

PARAMETER	SCORE			
	6	3	1	0
IC_50_ WT PfPKG (μM)	≤0.1	0.1< × <0.5	≥0.5	
IC_50_ HuPKGIα/WT PfPKG		>1000	10< × <1000	<10
HepG2 (μM)		100	50≤ × <100	<50
LE^a^		≥0.3	<0.3	
LLE^b^		>5	<5	
IFI^c^		<5	5≤ × <10	≥10
Compounds/cluster		≥5	2< × <4	1

^a^Ligand efficiency

^b^Lipophilic ligand efficiency

^c^Inhibition Frequency Index

Data mining on the prioritized scaffolds was performed and a summary of the general profile of each series can be found in the Supplementary Table 1. A safety profile was also performed *in silico*, which predicted that some compounds may be positive in the Ames test or contain a free aromatic amine, which renders the compounds toxic. However about 10% of the compounds are alert-free and ~50% may have minor alerts. Some scaffolds have not been previously described and may be good starting points for medicinal chemistry programs.

### The hits comprise new and known PKG inhibitors

Our initial hit confirmation studies focused on clusters 1–4, which display the tri-substituted heterocycle common structural feature. This scaffold has been previously described as an inhibitor of PKG and other plasmodial and coccidian kinases^11–13,32^. Among the four heterocycles, those containing the thiazole core were selected for full hit expansion activities, because information available from previous drug discovery programs describe this core as responsible for improved selectivity over human kinases^33^.

Taking advantage of the availability of more than 3,000 thiazole derivatives among the GSK compound collection, cluster 3 was thoroughly investigated for potent and selective inhibitors of WT PfPKG. The 3,180 available compounds were tested at a single concentration of 100 nM against the recombinant enzyme in a Kinase Glo assay and 350 compounds were identified that displayed more than 90% inhibition and were progressed to dose response and selectivity studies. Several compounds were identified which displayed potencies against WT PfPKG in the nanomolar range and selectivity against the human PKG orthologue of over 3 orders of magnitude. We explored the binding mode by also testing compounds on the gatekeeper mutant enzyme TQ PfPKG. The lack of inhibition of the mutant enzyme demonstrated that compounds interact with the gatekeeper pocket. The 32 most potent compounds were progressed to *P. falciparum* whole cell activity studies at 48 and 72 hours, where they displayed mid-nanomolar potencies (Supplementary Table 2). These two time points were chosen to give an indication of the speed of kill of the compounds. PKG-selective compounds have a relatively slow speed of kill which leads to a pronounced elevation of EC_50_ values in the 48 hour assay. A preliminary safety test was performed on human AuroraB and LCK kinases which often underpin toxicity issues. Results show that all compounds, with a few exceptions, have at least a 50-fold higher IC_50_ value against these human kinases than WT PfPKG. Further assays for compound characterization included determination of solubility and cytotoxicity (HepG2 inhibition). Thiazoles have been previously identified as human BRaf inhibitors that are used to treat melanoma^33^.

Importantly, although almost all compounds interact with the PKG gatekeeper pocket, most of the EC_50_ values in whole cell assays were very similar in WT parasites and TQ parasites indicating that, apart from PKG, these compounds have at least one other major target in blood stage parasites. Furthermore, the EC_50_ values were similar in the 48 hour and 72 hour assays, which is usually indicative of a fast kill compound. Selective PKG inhibitors have a moderate-to-slow kill mode of action, likely due to the involvement of PKG during the very narrow temporal window that encompasses merozoite egress and invasion^11^. The results suggest that the fast kill property of the thiazoles identified in this study is conferred by the non-PKG additional target(s).

### Chemoproteomics identifies additional targets of the thiazoles

**Figure 4:**
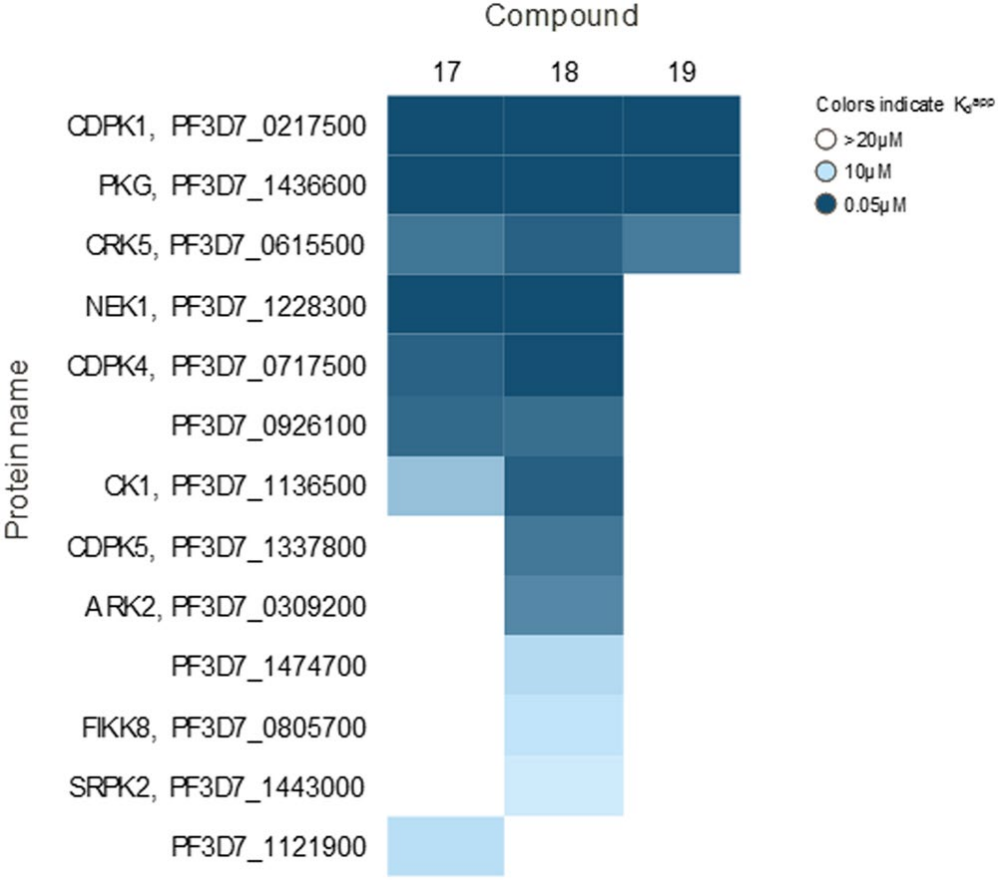
Chemoproteomic profiling on Kinobeads. This was performed using a *P. falciparum* protein extract to identify kinase targets for Compound 17, Compound 18 and Compound 19. The compounds were tested for their activity against 48 *P. falciparum* protein kinases and IC_50_ values were generated. The heat map shows all *P. falciparum* protein kinases affected by any of the 3 compounds (maximum compound concentration analysed was 20 µM, colours indicate apparent Kd values).

To identify potential additional kinase targets we explored a chemoproteomic approach: 3 thiazoles from the GSK compound collection (structures shown in Table 2) were analyzed on Kinobeads, which comprise a combination of immobilized promiscuous ATP-competitive kinase inhibitors^34,35^, to affinity capture potential kinase target proteins from a *P. falciparum* protein extract. The Kinobeads pull-down experiments were performed in the absence or presence of excess compounds 17, 18 and 19 to delineate kinase target proteins for which capture is competitively inhibited (Fig. 4). The experiments were performed by adding the compounds over a range of concentrations in order to establish a competition-binding curve and determine a half-maximal inhibition (IC_50_) value. The IC_50_ values obtained in these experiments represent a measure of target affinity, but are also affected by the affinity of the target for the bead-immobilized ligands. The latter effect can be deduced by determining the depletion of the target proteins by the beads, such that apparent dissociation constants (K_d_^app^) can be determined, which are largely independent from the bead ligand^36^. The proteins captured by the beads were quantified by isotope tagging of tryptic peptides followed by LC-MS/MS analysis^36^ (Supplementary Tables 5–7). All three of the analyzed compounds are potent inhibitors of recombinant PKG activity, but only two of these (Compounds 17 and 18) have strong activity in the 48 hour *P. falciparum* growth inhibition assay. Therefore kinases binding preferentially to these two compounds and not to the one with weak activity in the 48 hour assay (Compound 19), are more likely to be potential targets that could mediate fast killing of the parasites. Of the 50 *P. falciparum* kinases analysed on the Kinobeads, seven showed competition by both, Compound 17 and Compound 18. The activity against PKG was confirmed for all three compounds, with apparent dissociation constants (K_d_^app^) ranging between 50–60 nM. Two additional proteins were also competed by Compound 19 (the compound with weak activity): CDPK1 (K_d_^app^ 50–60 nM) and CRK5 (K_d_^app^ 1–3 µM). Four proteins were only affected by Compound 17 and Compound 18, but not by Compound 19: NEK1 (K_d_^app^ 50–120 nM), CDPK4 (K_d_^app^ 0.15–1.4 µM), CK1 (K_d_^app^ 1.2–7.7 µM) and an unnamed putative protein kinase Pf3D7_0926100 (K_d_^app^ 1.9–2.2 µM). Of the kinases that bind strongly to the thiazole scaffold, a global *P. falciparum* gene knockout study has indicated that PKG, CDPK1 and CK1 are essential whereas CDPK4, NEK1 and Pf3D7_0926100 are dispensable^37^. The mechanism of fast killing could potentially be due to the combined inhibition of PKG, and the above described additional kinases or to CK1, which is essential and only competed by the 2 compounds with activity against the parasite, although it is competed with high apparent K_d_ values.

**Table 2:** Structure and properties of the most balanced compounds identified in this study.

Compound			
	17	18	19
WT *Pf*PKG (µM)	0.0009	0.0008	0.0011
*Pf*PKG_T618Q (µM)	>1	0.5	>1
human PKG (µM)	>1	>1	>1
whole cell 48 h (µM)	0.18	0.19	3.4
whole cell 72 h (µM)	0.14	0.17	2.9
male/female (µM)	2.0/2.4	0.5/0.8	nd
HepG2 (µM)	79	50	39
Solubility^a^	73	142	116
ChromlogD^b^/PFI	1.3/5.3	1.6/5.6	1.9/5.9
pKa^c^	9.8	9.8	8.8

^a^Charged Aerosol Detector (CAD).

^b^ChromlogD measures lipophilicity.

^c^pKa measures the ionisation profile.

### The thiazoles show some promising properties

**Figure 5:**
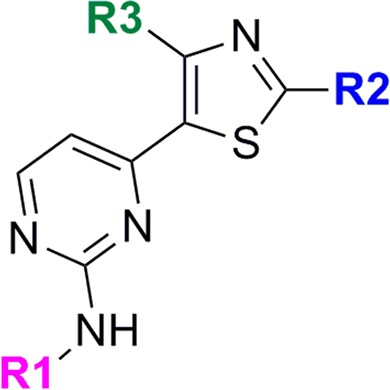
Common scaffold of cluster 3. R1, hydrogen; R2, alkyl; R3, substituted aromatic ring.

The shared scaffold of the thiazoles tested is shown in Fig. 5. Although these compounds have cytotoxicity and solubility issues, they are highly potent and selective (Supplementary Table 2). The R2 group is always an alkyl and R3 a substituted aromatic ring. The R1 group is always aromatic except in three cases, where R1 is a hydrogen. The three compounds shown in Table 2 have the best profile: lack of cytotoxicity, good solubility and specificity for WT PfPKG over the gatekeeper mutant (indicating that the compounds utilize the gatekeeper pocket). In addition, two compounds have nanomolar potency against blood stages and nanomolar to low micromolar potency against male and female gametocytes (Table 2). Compound 18 shows the most potent activity against male and female gamete formation (0.5 and 0.8 μM respectively). These results are consistent with our previous findings that PKG has an essential role in gametogenesis^13^. Additional work will be required to determine whether this activity against gamete activation is mediated solely by inhibition of PKG or whether the additional targets of the thiazoles are also important for this activity. Only in the case of Compound 19, the potency against blood stages is in the low micromolar range and the activities against the wild type enzyme and the parasite do not correlate, possibly due to its ionisation profile (pKa) and lipophilicity (ChromlogD). The thiazole compounds identified in this study represent valuable starting points for hit-to-lead progression.

## Discussion

PKG is involved in key *Plasmodium* erythrocytic stages, essential differentiation steps in transmission both to and from mosquitoes as well as in liver stage development. Having shown that PKG can be targeted successfully with small molecule inhibitors^11^, we sought to find additional scaffolds to add to the much-needed portfolio of possible future antimalarials. We performed high-throughput screening using the GSK Full Diversity Collection and found several scaffolds that provide a good starting point for the development of potent PKG inhibitors. Among the promising scaffolds are imidazopyridines that are reminiscent of the recently published series targeting PfPKG^11^ and of the imidazopyridazines, also shown to inhibit PfPKG^38^. The findings indicate that compounds containing an imidazole group are good scaffolds to inhibit PfPKG and also that HTS is a valid tool to identify inhibitors of this target. The analysis of safety alerts showed that most compounds have at least one or two safety alerts. This is expected when working with protein kinases, since they are ubiquitous and essential in most human cell functions. Knowledge of the safety alerts can drive the choice of the scaffolds to pursue chemical improvement. Moreover, the extensive knowledge of PfPKG and its recent structure solution will aid in optimizing compounds to target PfPKG^11^.

Of the newly identified scaffolds, compounds containing the thiazole moiety are the most promising series identified in our screen and similar compounds have previously been developed by GSK as BRaf kinase inhibitors. Dabrafenib is currently used in the clinic to treat melanoma caused by the gain of function mutant of BRaf kinase V600E^33^. Dabrafenib was shown to be highly selective for wild type and V600E BRaf when tested against a panel of 61 human kinases. In the clinic dabrafenib causes relatively mild toxicity, with only 24% of patients experiencing severe adverse events^39^. BRaf is a Ser/Thr kinase involved in the MAPK cascade. Interestingly, BRaf contains a Thr residue in the gatekeeper position, like PfPKG. Here, similarly to our earlier study, we found that PKG inhibitors utilize the gatekeeper pocket by showing that binding was abrogated in the TQ mutant PKG enzyme. The importance of Braf inhibition in a PKG-targeted antimalarial therapy will require additional studies. On the other hand, the acquired knowledge of BRaf SAR can be used to perform medicinal chemistry in the “opposite” way, that is, removing those chemical groups known to improve specificity for Braf, to avoid inhibiting BRaf and improve selectivity for PKG. A recent report using a template-hopping approach based on compounds that inhibit *Eimeria* PKG, has also shown that a series of trisubstituted thiazoles are effective inhibitors of the *P. falciparum* PKG^40^.

Protein kinases have been the focus of other antimalarial drug discovery screening studies. For example, Crowther *et al*. screened ~14,000 cell-active compounds against 5 *Plasmodium* kinases, with the aim of determining their molecular target^41^. They identified inhibitors of PK6, CDPK1 and CDPK4 but also found compounds that inhibit more than one target, similarly to our findings. Their findings suggest that it may be possible to develop a multi-kinase-targeting inhibitor that is safe in humans. Our findings using Kinobeads profiling of the thiazoles adds support to this notion. An inhibitor targeting PKG, that is essential throughout the life cycle, as well as another target that confers a fast speed of kill against blood stages would be a desirable profile. In another study, a phenotypic screen of a library of kinase inhibitors was performed on *P. falciparum* blood stages using a fluorescent assay to measure growth inhibition^42^. They identified nine interesting scaffolds and explored their selectivity using 76 mammalian kinases (that represent 15% of the mammalian kinome). Although there was excellent selectivity, they concluded that additional studies (such as protein kinase profiling or generation of resistant mutants) should be performed to determine whether parasite inhibition is due to one (or more) kinase.

PKG is highly conserved in all human malaria species. The overall sequence identity is 90–92% between *P. falciparum*, *P. vivax*, *P. malariae*, *P. ovale* and *P. knowlesi*, but residues in their catalytic sites, including the small gatekeeper residue, are identical^11^. In addition, crystal structures of the kinase domains of *P. falciparum* and *P. vivax* PKG superimpose almost perfectly^11^. Therefore, it is likely that a compound that blocks PfPKG also blocks PKG in other malaria parasite species, which makes a PKG inhibitor potentially a pan-malaria species drug. This is very important considering the high morbidity caused by *P. vivax* in the world and the alarming increase of infections of the zoonotic *P. knowlesi* parasite in South East Asia^1,43^.

In the landscape of kinase inhibitors as antimalarial drugs, the most advanced are compounds that target a phospholipid kinase, phosphatidylinositol-4 kinase (PI4K). The ATP competitive PI4K inhibitor MMV390048 gave rise to 0.57 and 1.1 mg/kg ED_90_ values against *P. falciparum* and *P. berghei* mouse models. It showed a clean selectivity profile and transmission blocking activity, with low micromolar potency against gametocytes. It also had prophylactic activity against *P. cynomolgi*, through prevention of liver stage development^44^. Another PI4K inhibitor, KDU691, showed activity against dihydroartemisinin-pretreated rings, which would provide a tool to combat dormant rings that linger in humans after artemisinin treatment^45^. Inhibition of phosphatidylinositol-3 kinase γ (PI3Kγ) with AS605240 delayed lethality in *P. berghei* ANKA infected mice and absence of PbPI3Kγ partially protected from experimental cerebral malaria^46^. Regarding protein kinases, apart from PKG, bumped kinase inhibitors of PfCDPK4 were developed as transmission blocking reagents. Interestingly, some of these also showed low micromolar potency against PfPKG^47^. Inhibitors of MEK1/2 (involved in the MAPK cascade) blocked severe malaria pathogenesis by regulating the immune response to *P. berghei* ANKA infection^48^. Finally, inhibition of the human Syk tyrosine kinase prevented phosphorylation of erythrocyte band 3 and consequently blocked egress in *P. falciparum*^49^.

More than 8 million compounds from several organizations have been screened for whole cell activity against *P. falciparum*, efforts that have delivered an enormous amount of starting points for basic research and drug discovery efforts. Currently, there is a well-populated antimalarial pipeline in the preclinical and clinical space with molecules such as the ozonide Artefenomel (Sanofi), the spiroindolone Cipargamin (Novartis), the imidazolopiperazine KAF156 (Novartis) and the triazolopyrimidine DSM265 (Takeda) (www.mmv.org/research-development/mmv-supported-projects). Interestingly, from all the pipeline opportunities, there are very few target-based programs, the DHODH inhibitors DSM265 and DSM411 being the most advanced assets in clinical development. Our study has identified promising starting points that can be improved through medicinal chemistry efforts, which will be aided by the extensive knowledge about the target and the availability of its crystal structure.

## Methods

### Recombinant enzyme production

Protein production was outsourced to the Division of Signal Transduction Therapy, University of Dundee. Briefly, the pTRChis expression vectors (Invitrogen) containing an N-terminally His6-tagged PfPKG wildtype and T618Q were used to transform BL21 cells (protocol adapted from^23^). The cells were grown at 37 °C until the OD_600_ was 0.4–0.6 then the temperature was reduced to 15 °C and the cells were induced with 250 µM IPTG and grown for a further 16–18 hours before harvesting. The His6-PfPKG was purified on Cobalt agarose followed by gel filtration and the protein was dialysed into 25 mM Hepes pH 7.5, 0.1 mM EGTA, 150 mM NaCl, 50% glycerol and 2 mM DTT.

The expression vector encoding His6-cGK1α (HuPKGIα) was used to generate recombinant baculoviruses using the Bac-to-Bac system (Invitrogen) following the manufacturer’s protocol. These baculoviruses were used to infect *Spodoptera frugiperda* 21 cells (1.5 × 106/ml) at a multiplicity of infection of 5 and the infected cells were harvested 48 hours post-infection. His6-cGK1α was purified on Cobalt agarose and dialysed into 50 mM Tris-HCl pH 7.5, 0.1 mM EGTA, 150 mM NaCl, 270 mM sucrose, 0.03% Brij-35, 0.1% 2-mercaptoethanol, 1 mM benzamidine, 0.1 mM phenylmethylsulfonyl fluoride (PMSF).

### Optimisation of the PKG assay for high-throughput screening

Initial optimization of the PKG assay was performed manually in 384-well plates and analysed with GraFit 7. The buffer for all assays was 25 mM HEPES pH 7.3 (GIBCO), 10 mM MgCl_2_ (Sigma), 2 mM DTT (Sigma), 0.01 mM cGMP (Sigma), 0.1 mg/ml BSA (Ambion). Enzymatic activity was tested using a luminescence-based kit (Promega KinaseGlo). Addition of the first reagent depletes the unused ATP and addition of the second reagent converts ADP (product of the kinase reaction) to ATP, which is then measured through a luciferin/luciferase reaction. The amount of luminescence is directly proportional to ATP turnover by the kinase. The kit was chosen because it can be miniaturized to small volumes (i.e. 4 µl) in 1536-well plates, for use in HTS. We performed all assays using an incubation time of 1 hour and 0.5 nM WT PfPKG, TQ PfPKG and HuPKGIα because these conditions resulted in a signal to background (that is luminescence signal divided by control 1, no enzyme) values equal to 11.3, 8.6 and 19.8 respectively.

We used PKA peptide substrate (SCP0212, Sigma-Aldrich) for WT and TQ PfPKG, dissolved in milliQ water at a final concentration of 5 mM, and Long S6 peptide substrate (produced at the University of Dundee, sequence KEAKEKRQEQIAKRRRLSSLRASTSKSGGSQK) for HuPKGIα, dissolved in water at a final concentration of 30 mM.

We calculated the Michaelis-Menten (K_m_) constant for ATP and peptide substrate (PKA or Long S6) for all enzymes using GraphPad Prism (Supplementary Figs 2 and 3 and Supplementary Table 3). In order to identify different types of PKG inhibitors, including ATP competitive or non-competitive inhibitors, as well as inhibitors that bind to the peptide substrate pocket, we performed all assays using ATP and peptide substrate at a concentration equal to the K_m_. We also determined enzyme activity by calculating ATP turnover (K_cat_) over the K_m_ (Supplementary Table 3). The K_cat_ was calculated as (V_max_*slope)/[E] where V_max_ is the maximum velocity and [E] is the concentration of the enzyme.

Enzymatic activity was not affected by DMSO, which was used at 1% in the assay (Supplementary Fig. 4). Cyclic GMP was used in excess (10 mM) (data not shown). PfPKG is known to undergo autophosphorylation^23^ but it was decided to not take into account the contribution of autophosphorylation because it represented only ~1% of the total signal (data not shown).

### High-throughput screening

The plates containing the compounds were prepared at GSK in Upper Providence or Stevenage. Compounds were tested at a single final concentration of 10 µM or in a dose response with a range of concentrations starting from 100 μM to 1.6 nM (1 in 3 serial dilutions). Compounds were dispensed in the plates at the required concentration in a 40 nl volume. All plates (1536-well white Greiner) contained 40 nl of DMSO in lanes 11 and 12 (control 1, 100% enzyme activity) and were empty in lanes 35 and 36 (control 2, 0% enzyme activity). The plates were removed from −20 °C 1 hour before starting the assay. Using a Multidrop Combi dispenser (Thermo Scientific), 2 µl of the buffer containing ATP and the substrate were added to all wells then 2 µl of buffer only were added to column 35 and 36. Finally 2 µl of buffer containing the enzyme were dispensed in all the plate wells except for lanes 35 and 36. After 1 hour incubation at 21 °C 2 µl of ADP-Glo reagent (Promega KinaseGlo kit), previously supplemented with 0.02% CHAPS (Sigma), were added to all wells. After 40 minutes incubation at 21 °C, 2 µl of Kinase-Glo reagent (Promega KinaseGlo kit), previously supplemented with 0.02% CHAPS (Sigma), were added to all wells. After 30 minutes incubation at 21 °C in the dark the resulting luminescence was read at a Perkin Elmer ViewLux with the following settings: clear filter, 5 seconds exposure time, slow speed, 50x gain, 2x binning. The HTS was carried out in 14 days with an average of 128 plates analysed per day. Each set of plates that was tested also included one plate of tool compounds (see below) as a positive control.

To discard luminescent compounds that would interfere with the assay’s read out, we set up a Counterscreen assay. We calculated the amount of ADP that WT PfPKG usually produces in an assay (0.82 μM) and we used this concentration to mimic the enzyme’s activity, without adding enzyme or substrate. Compounds that showed a change in signal were considered luminescent and discarded.

The HTS, Confirmation and Dose-Response results were analysed using Activity Base XE (IDBS, Guilford, Surrey, UK) and were displayed using Spotfire (TIBCO Spotfire^®^). The results of the parasite assays were analysed by Excel-XLFit and displayed using Spotfire.

Percentage of inhibition was calculated as 100*(Lum_well_ – Lum_ctr1_)/(Lum_ctr2_ – Lum_ctr1_).

The cutoff was calculated as (Mean % inhibition)*(3xStandard Deviation).

### Tool compounds

First a set of tool compounds was tested to check that the inhibition assay worked well. The tool compounds are a set of known kinase and PfPKG inhibitors (Supplementary Table 4 and data not shown). They were tested in duplicate against WT PfPKG, TQ PfPKG and HuPKGIα. Results showed that the IC_50_ values were similar to the published ones^23,50^, with only a few differing by more than one order of magnitude, probably due to the different preparations of the enzyme or assay conditions between laboratories. We also included compounds that were identified previously^11^. As expected, these did not inhibit TQ PfPKG (or inhibited it but with more than 2 orders of magnitude of difference) because they are inhibitors that utilize the gatekeeper pocket to inhibit WT PfPKG.

### Compound prioritization through scoring system

A scoring system was developed to further prioritize interesting compounds. Highest weighting was given to the most potent compounds by giving six points to those that inhibited WT PfPKG with an IC_50_ of ≤0.1 μM. Three points were given to compounds that inhibit HuPKGIα at least 3 orders of magnitude less than WT PfPKG. Compounds that showed low cytotoxicity (EC_50_ with HepG2 ≥ 100 μM) were given 3 points. We also considered how well the compounds bind to the target by taking into account two physicochemical properties: ligand efficiency (LE, 1.37 x pIC_50_/number heavy atoms) is a characterization of a compound’s efficiency on a per-heavy-atom basis^51^ and lipophilic ligand efficiency (LLE, pIC_50_ – clogP) is a parameter used in drug design which links potency and lipophilicity in an attempt to estimate drug-likeness^52^. The ClogP coefficient measures lipophilicity. Values of LE ≥ 0.3 and LLE ≥ 5 are typically accepted as good starting points for optimizations efforts. To study the promiscuity profile of the compounds, the Inhibition Frequency Index (IFI, number of HTS assays where a compound showed >50% inhibition/Total number of HTS assays) was calculated^7^. Lastly, clusters containing numerous compounds were prioritized due to the estimated better SAR possibilities.

### Whole cell assay

Parasite growth inhibition assays were performed as previously described^7^. *P. falciparum* clone 3D7A were kept at 2% haematocrit in RPMI-1640, 5% AlbuMAX, 2% d-sucrose, 0.3% glutamine and 150 µM hypoxanthine. Ring stage parasites were incubated with 50 nl of the compounds in a 384-well plate (clear bottom dark Greiner) for 48 or 72 hours in a 5% CO2, 90% N2 and 5% O2 atmosphere. Plates were frozen at −80 °C over night and thawed for 3 hours before starting the assay. A solution to measure the activity of parasite LDH was prepared (143 mM sodium L-lactate, 143 μM 3-acetyl pyridine adenine dinucleotide (APAD), 178.75 μM Nitro Blue tetrazolium chloride (NBT), 286 μg/ml diaphorase (2.83 U/ml), 0.7% Tween 20, 100 mM Tris-HCl pH 8.0) and incubated for 10 minutes at 21 °C before reading absorbance at 650 nm in a spectrophotometer. Artesunate at a final concentration of 4 μM was used as a positive control, while DMSO was used as a negative control. Compounds for whole cell assays were tested at a single final concentration of 2 µM or in a dose response with a range of concentrations between 5 or 10 μM to 0.08 nM (1 in 3 serial dilutions).

### Dual gamete activation assay

This bioassay assesses the malaria transmission blocking potential of compounds by estimating their ability to prevent male mature gametocytes progressing to male microgametes and/or to inhibit female gamete activation, as indicators of gametocyte functionality. Activation of male gametocytes to form mature microgametes is evaluated by the process of exflagellation (extrusion of highly motile microgametes from the infected erythrocyte). The activation of female gametocytes is evaluated based on the specific expression of the Pfs25 protein at the surface of the female gametes.

Gametocyte cultures were produced as follows: asexual cultures of *P. falciparum* (NF54 isolate) parasites were used to seed gametocyte cultures at 0.5% parasitemia, 4% hematocrit in a 50 ml total volume under 3% O_2_/5% CO_2_/92% N_2_ gas. Culture medium (RPMI 25 mM HEPES, 50 mg/l hypoxanthine, 2 g/l NaHCO_3_ + 5% human serum and 5% Albumax) was replaced daily for 14 days. At day 14, the concentration of unpurified cultures was adjusted to plate 700,000 total cells per well in each 384-well plate. The test drugs were then added and incubated 48 h at 37 °C (3%0_2_, 5%CO_2_, 92%N_2_). Gametocyte activation was then triggered by a drop in temperature and addition of xanthurenic acid (D120804, Sigma) as previously described^53,54^. To detect female gametes, monoclonal anti-Pfs25 antibody38 4B7 (BEI Resources (formerly MR4), cat. no. MRA-315) conjugated to the Cy3 fluorochrome (GE Healthcare) was added to ookinete medium (RPMI 1640 supplemented with 25 mM HEPES, 50 mg/ml hypoxanthine, 2 g/l NaHCO_3_, 100 mM xanthurenic acid and 20% human serum) at a final concentration of 0.5 mg/ml.

Immediately after activation, the plates were incubated at 26 °C for 3–5 min and detection of exflagellation centres was performed in an automated inverted microscope Ti-E Nikon using bright field. Plates were then incubated (protected from light) at 26 °C for 24 hours. Plates were then analyzed to detect Pfs25 expression in an automated inverted microscope Ti-E Nikon using TRITC fluorescence.

For activation of male gametes, detection is based on light changes provoked by flagellar movements which cause movement of surrounding cells. A 10-frame video is taken and then analyzed to determine these changes in cell position based on pixel changes. The script determines where exflagellation centers are located, based also on the size and intensity of light changes. For activation of female gametes, detection of fluorescent Cy3-Anti Pfs25 antibody (as primary parameter) is performed followed by selection of events according to their size, roundness and the intensity of the fluorescence. Both measurements are performed using an automated inverted microscope Ti-E Nikon using JOBS software. Analysis of images and videos is performed with ICY program.

### Cytotoxicity assay

Cytotoxicity assays were performed on HepG2 cells as previously described^55^. HepG2 cells were removed from a T-175 TC flask using 5 mL Eagle’s MEM (containing 10% FBS, 1% NEAA, 1% penicillin/streptomycin) and dispersed in the medium. Seeding density was checked to ensure that new mono-layers were not more than 50% confluent at the time of harvesting. Cells were added to 500 mL of the same medium at a final density of 1.2 × 10^5^ cells/mL. They were then dispensed (25 μL, about 3,000 cells per well) into 384-well clear-bottom plates using a Multidrop Combi dispenser. Test compounds (250 nL) were dispensed into the plates with an EchoH liquid handler prior to addition of the cells. Plates were incubated for 48 h at 37 °C, 5% CO_2_. After incubation, plates were equilibrated at room temperature for 30 minutes before proceeding to develop the luminescent signal. The CellTiter-GloH Reagent was allowed to equilibrate at room temperature for 30 minutes and added to the plates (25 μL per well) using a Multidrop Combi dispenser. Plates were left for 10 minutes at room temperature for stabilization and then read using a Perkin Elmer ViewLux.

REF1: Safety predictions were performed using the following Software packages: Leadscope v2.2.1 (Salmonella v3, E.Coli-Sal102 v1), DEREK Nexus v6.0 (2018 KB v1.1), eHOMO (Energy of the Highest Occupied Molecular Orbital).

### Aqueous kinetic solubility assay

5 ml of 10 mM DMSO compound solution is diluted to 100 μl with pH7.4 phosphate buffered saline, equilibrated for 1 hour at room temperature and filtered through Millipore Multiscreen_HTS_-PCF filter plates (MSSL BPC). The filtrate is quantified by suitably calibrated Charged Aerosol Detector^56^. The upper limit of the solubility is 500 µM when working from 10 mM DMSO stock solution.

### Chemoproteomics

Kinobeads were prepared as described^34–36^ and the chemoproteomic affinity capturing experiments were performed as previously described^36^. Briefly, Kinobeads were washed and equilibrated in lysis buffer (50 mM Tris-HCl, pH 7.4, 0.4% Igepal-CA630, 1.5 mM MgCl_2_, 5% glycerol, 150 mM NaCl, 25 mM NaF, 1 mM Na_3_VO_4_, 1 mM DTT, and one Complete EDTA-free protease inhibitor tablet (Roche) per 25 mL). The beads were incubated at 4 °C for 1 h with 0.1 mL (0.3 mg) *P. falciparum* extract, which was pre-incubated with compound or DMSO (vehicle control). The experimental set up was such that 10 samples are measured in parallel (TMT 10-plex^57^, to generate values for the affinity of the beads to the bound proteins (“depletion” values, 4 samples) and to generate IC_50_ values (6 samples) in a single experiment. Samples 1 and 2 were the vehicle control, samples 3 and 4 were processed in the same way, but while the beads were discarded after the first incubation step the extract was incubated with fresh beads to measure how much protein could rebind to the fresh beads (was depleted from the extract by first bead-binding). Apparent dissociation constants were determined by taking into account the protein depletion by the beads^36^. Samples 5–10 were used to generate IC_50_ values by adding compound over a range of concentrations (20 µM, 1:3 dilutions). Beads were transferred to filter plates (Durapore (PVDF membrane, Merck Millipore), washed extensively with lysis buffer and eluted with SDS sample buffer.

Proteins were digested according to a modified single pot solid-phase sample preparation (SP3) protocol^58,59^. Briefly, proteins in 2% SDS were bound to paramagnetic beads (SeraMag Speed beads, GE Healthcare, CAT#45152105050250, CAT#651521050502) by addition of ethanol to a final concentration of 50%. Contaminants were removed by washing 4 times with 70% ethanol. Proteins were digested by resuspending in 0.1 mM HEPES (pH 8.5) containing TCEP, chloracetamide, trypsin and LysC following o/n incubation.

Peptides were labeled with isobaric mass tags (TMT10, Thermo FisherScientific, Waltham, MA using the 10-plex TMT reagents, enabling relative quantification of 10 conditions in a single experiment^57,60^. The labeling reaction was performed in 40 mM triethylammoniumbicarbonate, pH 8.5 at 22 °C and quenched with glycine. Labeled peptide extracts were combined to a single sample per experiment, lyophilized and subjected to LC-MS analysis.

Dried samples were resuspended in 0.05% trifluoroacetic acid in water. Half of the sample was injected into an Ultimate3000 nanoRLSC (Dionex) coupled to a Q-Exactive (Thermo Fisher Scientific). Peptides were separated on custom-made 35 cm × 100 μm (ID) reversed-phase columns (Reprosil) at 55 °C, gradient elution was performed from 3.5% acetonitrile to 29% acetonitrile in 0.1% formic acid and 3.5% DMSO over 120 minutes.

Samples were online injected into a Q Exactive Plus mass spectrometer. The Q Exactive Plus was operating with data-dependent top 10 method. MS spectra were acquired using 70.000 resolution and an ion target of 3E6. Higher energy collisional dissociation (HCD) scans were performed with 35% NCE at 35.000 resolution (at m/z 200), and ion target settings was set to 2E5 so as to avoid coalescence^57^. The instruments were operated with Tune 2.4 and Xcalibur 3.0 build 63.

Mascot 2.4 (Matrix Science, Boston, MA) was used for protein identification by using a 10 parts per million mass tolerance for peptide precursors and 20 mD (HCD) mass tolerance for fragment ions. To create the fasta file for mascot searching, all proteins corresponding to the taxonomy ‘*Plasmodium falciparum* (isolate 3D7)’ were downloaded from Uniprot (release 20170621) and supplemented with common contaminant protein sequences of bovine serum albumin, porcine trypsin and mouse, rat, sheep and dog keratins. To assess the false discovery rate (FDR) “decoy” proteins (reverse of all protein sequences) were created and added to the database, resulting in a database containing a total of 14266 protein sequences, 50% forward, 50% reverse.

Carbamidomethylation of cysteine residues and TMT modification of lysine residues were set as fixed modifications. Methionine oxidation, N-terminal acetylation of proteins and TMT modification of peptide N-termini were set as variable modifications.

Unless stated otherwise, we accepted protein identifications as follows: (i) For single-spectrum to sequence assignments, we required this assignment to be the best match and a minimum Mascot score of 31 and a 10 × difference of this assignment over the next best assignment. Based on these criteria, the decoy search results indicated <1% false discovery rate (FDR). (ii) For multiple spectrum to sequence assignments and using the same parameters, the decoy search results indicated <0.1% FDR. Quantified proteins were required to contain at least 2 unique peptide matches. FDR for quantified proteins was <0.1%. Raw data tables for the chemoproteomics experiments can be found in the Supplementary Tables 5–7.

I certify that the research using each of the HBS marked above was conducted according to the requirements of POL-GSKF-410 and associated relevant SOPs, and that all related documentation is stored in an approved HBSM database.

The human biological samples were sourced ethically and their research use was in accord with the terms of the informed consents under an IRB/EC approved protocol.

## Supplementary information

Supplementary Information

Supplementary Table 1

Supplementary Table 2

Supplementary Table 5

Supplementary Table 6

Supplementary Table 7

## Data Availability

All data generated or analysed during this study are included in this published article (and its Supplementary Information files).
