# Supplementary material for: High-throughput screening of the Plasmodium falciparum cGMP-dependent protein kinase identified a thiazole scaffold which kills erythrocytic and sexual stage parasites
Source: Sci Rep. 2019 May 7;9:7005. doi: 10.1038/s41598-019-42801-x (PMC6504873; doi:10.1038/s41598-019-42801-x)
Supplement: Supplementary file 1 — Supplementary Information [file 41598_2019_42801_MOESM1_ESM.pdf]

Combined Supplementary data

(See also Supplementary datasets Tables 1, 2, 5-7)

High-throughput screening of the *Plasmodium falciparum* cGMP-dependent protein kinase identified a thiazole scaffold which kills erythrocytic and sexual stage parasites

Maria Penzo, Laura de las Heras-Dueña, Lydia Mata Cantero, Beatriz Diaz-Hernandez, Maria-Jesus Vazquez-Muñiz, Sonja Ghidelli-Disse, Gerard Drewes, Elena Fernandez-Alvaro, David A. Baker.

Supplementary Figure 1: Correlation of the confirmation step tested in duplicate against HuPRKGIα

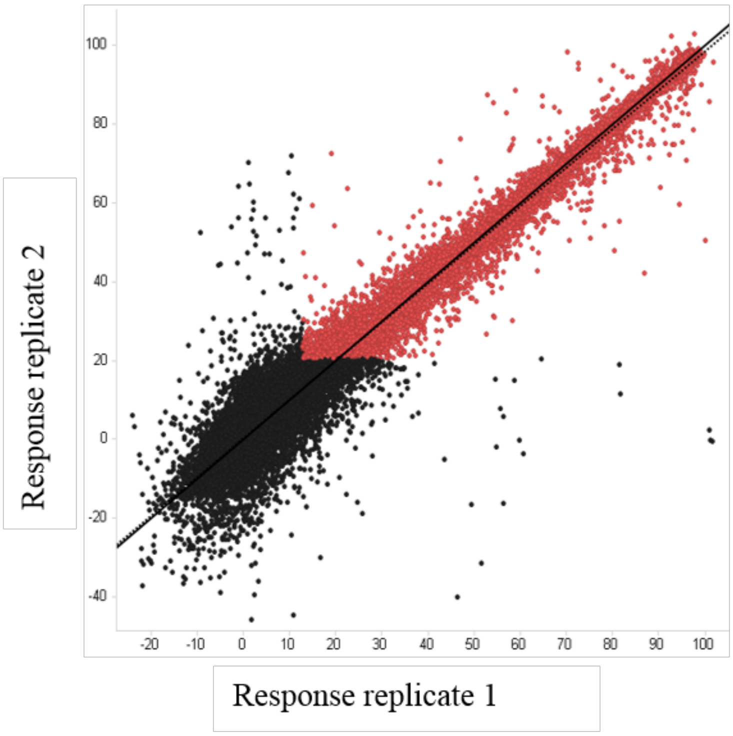

Supplementary Figure 2: Determination of the K_m for ATP

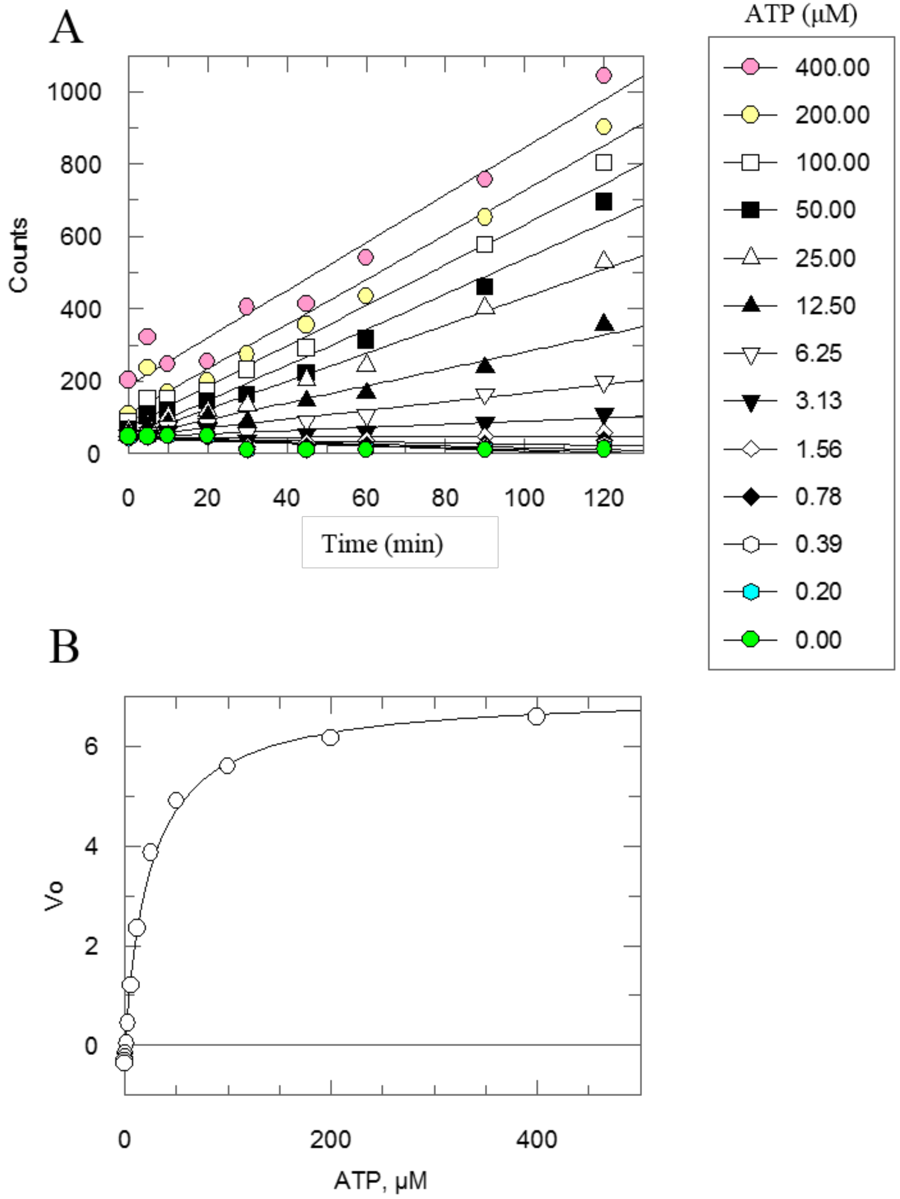

Parameter	Value	Std. Error
Vmax	7.0624	0.2644
Km	24.9965	3.4213

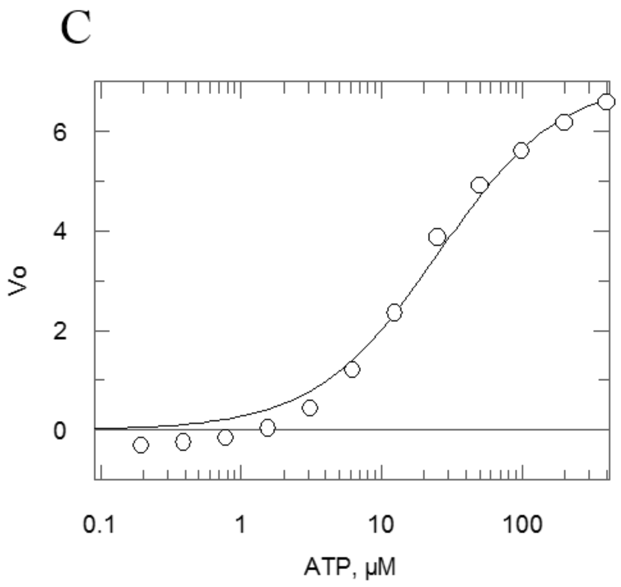

Supplementary Figure 3: Determination of the K_m for PKA peptide substrate

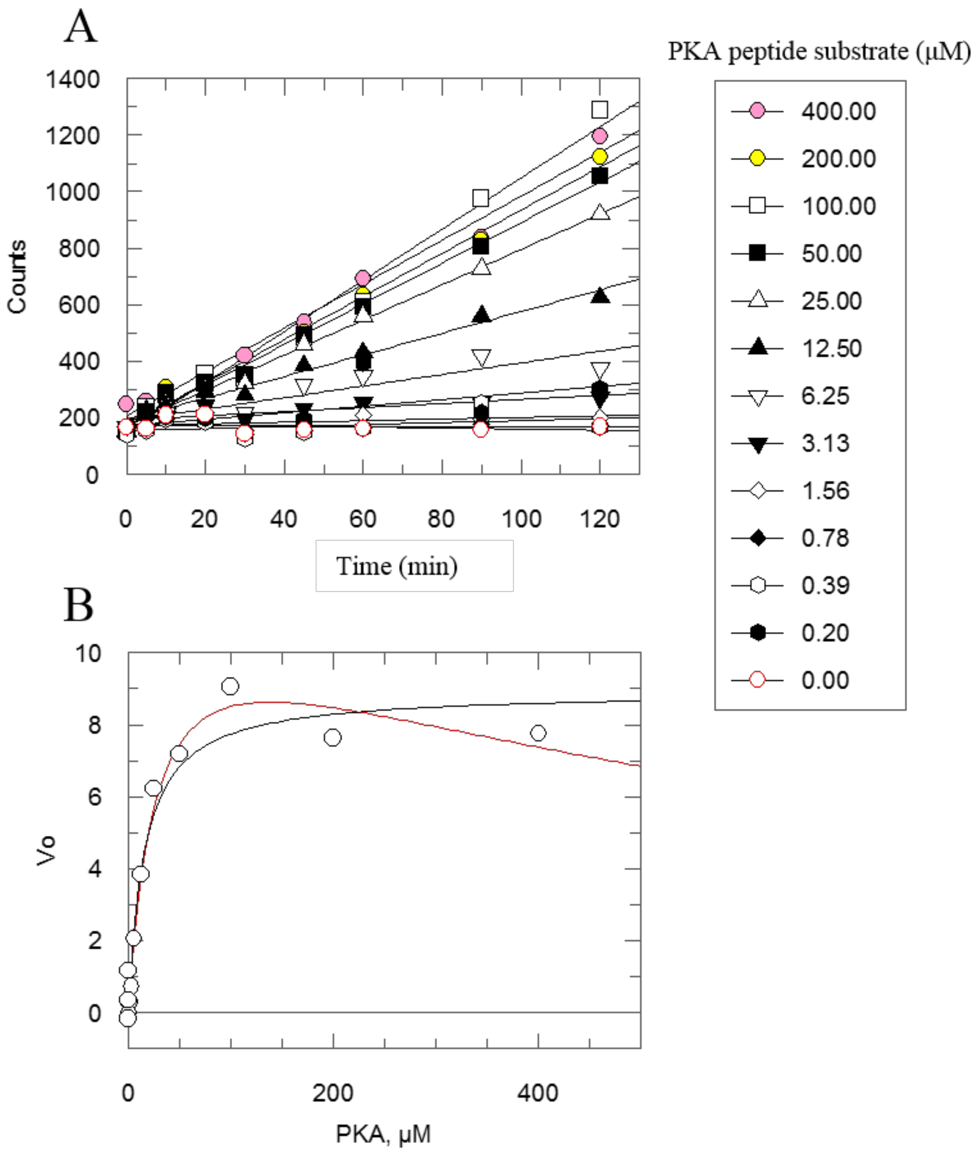

Parameter	Value	Std. Error
Km	26.5677	7.6679
Ki	723.7427	352.2657
Vm	11.9369	1.6221

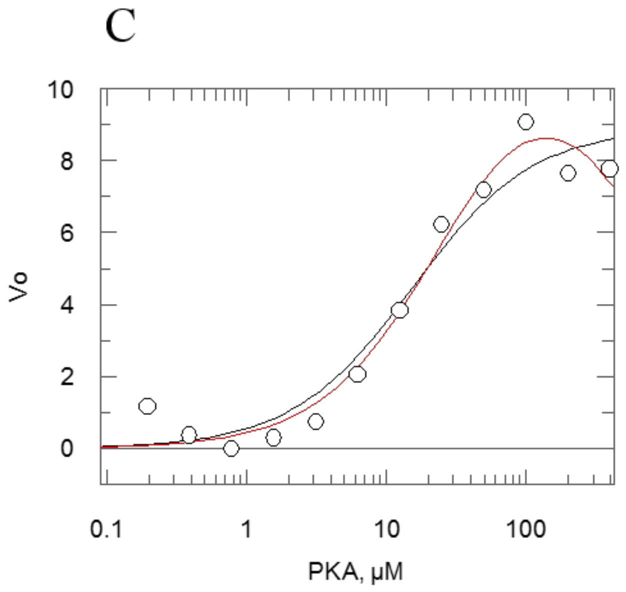

Supplementary Figure 4: DMSO does not affect enzyme activity at low percentages

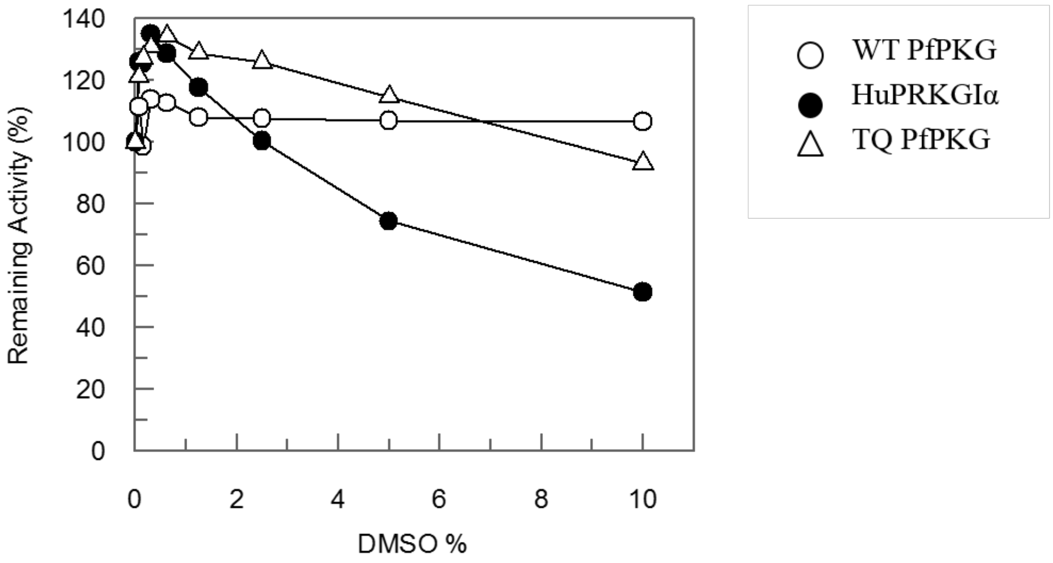

Supplementary Table 3: Kinetic data for recombinant PKG enzymes

	**WT PfPKG**	**TQ PfPKG**	**HuPKGIα**
K_m ATP (μM)	25 ± 3	82 ± 6	42 ± 1.5
K_m substrate (μM)	PKA: 26.5 ± 7	PKA: 38 ± 7	S6: 41 ± 12
K_{cat} (min^{-1})	324	274	688
K_{cat}/K_m	12.5	7.2	16.8

Supplementary Table 4: Relative potency of tool compounds against recombinant PKG enzymes

	**Known IC_{50} WT PfPKG (µM)**	**Known IC_{50} TQ PfPKG (µM)**	**IC_{50} WT PfPKG (µM)**	**IC_{50} TQ PfPKG (µM)**	**IC_{50} HuPKGIα (µM)**	**Reference**
ML1[a]	0.003	7.943	0.002	5.012	> 100	Baker et al. 2017
GF109203X[b]	0.079 (PKN1)[c]	-	0.631	1.0	1.0	Falk et al. 2014
Staurosporine[b]	0.010	-	0.003	0.025	0.006	Deng et al. 2002

[a] Known PfPKG inhibitor
[b] Known kinase inhibitors
[c] Kinase on which the IC_{50} was calculated, when not PfPKG

Supplementary Information Legends

Figure S1: Correlation of the confirmation step tested in duplicate against HuPRKGIα. Compounds in red are above the cut-off of 30% inhibition.

Figure S2: Determination of the Michaelis-Menten constant (K_m) for ATP. The enzymatic reaction was performed at saturating concentrations of PKA peptide substrate (400 µM). Panel A shows the linear progression of the reaction. Dots of different size and colour represent ATP concentration (µM). Panel B and C show the initial velocity (V_0) plotted against the ADP formed in the enzymatic reaction in respectively linear and logarithmic scale. The table shows the calculated V_{max} and K_m with the corresponding standard errors.

Figure S3: Determination of the K_m for PKA peptide substrate. The reaction was performed at saturating concentrations of ATP (400 µM). Panel A shows the linear progression of the reaction. Dots of different size and colour represent PKA peptide substrate's concentration. Panel B and C show the initial velocity (V_0) plotted against the ADP formed in the enzymatic reaction in respectively linear and logarithmic scale. At high PKA peptide substrate's concentrations the reaction is partly inhibited. This is shown by the inhibition curve in red. The table shows the calculated V_{max}, K_m and inhibition constant (K_i) with the corresponding standard errors.

Figure S4: DMSO does not affect enzyme activity at low percentages. Recombinant enzyme activity was titrated at varying DMSO concentrations. The remaining enzymatic activity was

calculated as (100*(signal-No DMSO)/(No enzyme-No DMSO)). The DMSO concentration in all screens performed was 1% therefore enzyme activity is not affected.

Table S1: Profile of the 66 compounds that had a score of at least 15.

Table S2: A potent series of thiazoles has nanomolar activity against *P. falciparum* parasites.

Table S3: Kinetic data for recombinant PKG enzymes.

Table S4: Relative potency of tool compounds against recombinant PKG enzymes.

Table S5: MS/MS data for profiling of Compound 17 on Kinobeads using a *P. falciparum* extract (human proteins quantified by MS/MS are also shown): 10 samples were analyzed in parallel (TMT 10-plex) to generate values for the affinity of the beads to the bound proteins ("depletion" values, 4 samples) and to generate IC_{50} values (6 samples) in a single experiment. Samples 1 and 2 represent the vehicle control, samples 3 and 4 were processed in the same way, but while the beads were discarded after the first incubation step the extract was incubated with fresh beads to measure how much protein could rebind to the fresh beads (was depleted from the extract by first bead-binding). Apparent dissociation constants were determined by taking into account the protein depletion by the Kinobeads. Samples 5-10 were used to generate IC_{50} values by adding compound over a range of concentrations (20 µM, 1:3 dilutions).

Table S6: MS/MS data for profiling of Compound 18 on Kinobeads using a *P. falciparum* extract (human proteins quantified by MS/MS are also shown): 10 samples were analyzed in parallel (TMT 10-plex) to generate values for the affinity of the beads to the bound proteins (“depletion” values, 4 samples) and to generate IC_{50} values (6 samples) in a single experiment. Samples 1 and 2 represent the vehicle control, samples 3 and 4 were processed in the same way, but while the beads were discarded after the first incubation step the extract was incubated with fresh beads to measure how much protein could rebind to the fresh beads (was depleted from the extract by first bead-binding). Apparent dissociation constants were determined by taking into account the protein depletion by the Kinobeads. Samples 5-10 were used to generate IC_{50} values by adding compound over a range of concentrations (20 µM, 1:3 dilutions).

Table S7: MS/MS data for profiling of Compound 19 on Kinobeads using a *P. falciparum* extract (human proteins quantified by MS/MS are also shown): 10 samples were analyzed in parallel (TMT 10-plex) to generate values for the affinity of the beads to the bound proteins (“depletion” values, 4 samples) and to generate IC_{50} values (6 samples) in a single experiment. Samples 1 and 2 represent the vehicle control, samples 3 and 4 were processed in the same way, but while the beads were discarded after the first incubation step the extract was incubated with fresh beads to measure how much protein could rebind to the fresh beads (was depleted from the extract by first bead-binding). Apparent dissociation constants were determined by taking into account the protein depletion by the Kinobeads. Samples 5-10 were used to generate IC_{50} values by adding compound over a range of concentrations (20 µM, 1:3 dilutions).
